# Health disparities research is enabled by data diversity but requires much tighter integration of collaborative efforts

**DOI:** 10.7189/jogh.10.020351

**Published:** 2020-12

**Authors:** Jean-Baptiste Cazier, Liudmila Sergeevna Mainzer, Weihao Ge, Justina Žurauskienė, Zeynep Madak-Erdogan

**Affiliations:** 1 Centre for Computational Biology, University of Birmingham, Edgbaston, Birmingham, UK; 2 Institute of Cancer and Genomic Sciences, University of Birmingham, Edgbaston, Birmingham, UK; 3 National Center for Supercomputing Applications, University of Illinois at Urbana-Champaign, Urbana, Illinois, USA; 4 Carl R. Woose Institute for Genomic Biology, University of Illinois at Urbana-Champaign, Urbana, Illinois, USA; 5 Department of Food Science and Human Nutrition, Division of Nutritional Sciences, University of Illinois at Urbana-Champaign, Urbana, Illinois, USA; 6 Cancer Center at Illinois, Beckman Institute for Advanced Science and Technology, University of Illinois at Urbana-Champaign, Urbana, Illinois, USA

The world is diverse, and this needs to be better recognized and addressed in health research. Health Disparities (HD) are a growing concern, which affects not only the world at a global scale, but individual countries and their own diversity [1]. The spectrum of individual health is moulded not solely by genetics or socio-economics [2], but by a combination of numerous factors, which include other key parameters such as geographic location [3] (to reflect rurality or segregation that can reduce access to care), impeding monitoring, risk reduction, diagnosis, and treatment of medical conditions [4]. The multifaceted nature of the problem demands availability of relevant data, analysis approaches, and research infrastructure. In addition to interdisciplinary partnerships among scientists in health, geography, data science or sociology, work is also needed to unite researchers, clinicians, politicians, and the communities themselves (**Figure 1**). Only such harmonious integration across stakeholders will ensure the impact of complex health data are accurate, useful, and actionable. Failure to accommodate the diversity of needs using equally diverse and relevant data results in HD.

**Figure 1:**
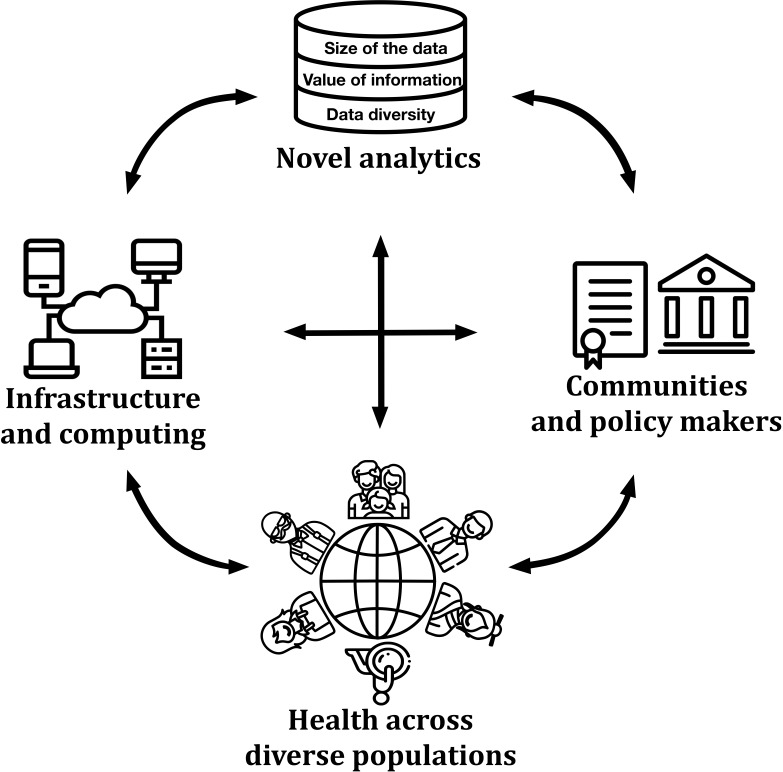
Enabling health disparities research. Committed collaboration is necessary between communities, scientists, infrastructure engineers, and policy makers to address Health Disparities. This can only be achieved through a cohesive interplay between the development of novel analytics approaches for very large, diverse data sets, advances in computational infrastructure for enhanced security, supportive policies in data sharing, and greater involvement of communities in decision making about their health. We are acknowledging the following www.flaticon.com vector icon artists: Eucalypt, turkkub, Good Warem, Freeic.

## DIVERSITY OF DATA

Data collection has always been underpinning public health research and policies [5]. Technology has made large scale multifactorial collection affordable and ubiquitous [6], enabling rapid progress in research and translation between populations, thus raising the prospect of next-generation data-driven health care. The clinical profile can now be enriched with a multi-omics signature, detailed lifestyle, and health markers from wearable devices, neighbourhood wealth, local pollution level, etc. However, the unequal access to such breadth and depth of continuous health related information is widening HD. Furthermore, limited return in actionable health improvement, combined with data becoming an important tradable commodity has led to an increased mistrust of efforts in health data collection: populations have begun to feel like a commodity rather than a beneficiary. Increased transparency and open collaboration with the community stakeholders are necessary to alleviate this problem. Fair usage of data should be enforced by transnational institutions and governments to address ethical issues, third-party access and re-identifiability. Methods and findings must be integrated and disclosed to regain public trust in research on diversity and HD.

## QUALITY CONTROL AND NOVEL ANALYTICS

Thorough HD studies require very large longitudinal data sets [7] to enable inclusion of all relevant factors while maintaining statistical significance. However, accumulating appropriate sample sizes is laborious and logistically complex, causing researchers to combine data from different collections. This causes problems with quality control: adjusting for differences among many independent protocols and management systems, as well as missing, incorrect and mismatching data. Importantly, the variety of sources reflecting population diversity can itself contribute to an additional disparity bias. International coordination between individual data sources is essential to define ontologies and improve usability of data thus ensuring reproducibility of HD research.

The inherent heterogeneity of HD data creates special challenges and opportunities related to analytical practices. HD data are multi-layered and hierarchically structured to reflect their range of sources [3]. Data are collected at the environmental/macro (geographical location, neighbourhood wealth, crime, pollution) and personal/micro (familial, socioeconomic, lifestyle, electronic health records, and wearables) levels. Further, -omics data are inherent in HD research to investigate the molecular level. Joint analysis of these data dimensions is a powerful way to unveil their interactions, potentially pinpointing causal mechanisms of HD, improving our understanding of disease manifestation and subtyping, and enabling appropriate intervention on a condition. However, analysis of such multi-level, multidimensional data sets is challenged by heterogeneity of variables (binary, categorical, continuous, semantic, with numerical values in disparate ranges), that require specific techniques for curation, aggregation, and analysis. There is a real need to extend existing approaches and build new tools that can integrate the large-scale, high-dimensional, and multisource data from vulnerable and underrepresented populations. This includes (a) addressing data multilayer-ness and polysemy between potential confounders and mediators [8], (b) capturing interactions between physiology and environment, and (c) improving statistical robustness of analyses to deliver confidence in predictions, condition identification and risk factors.

## INFRASTRUCTURE AND COMPUTING

Diversity, complexity, and privacy of the HD data sets pose unique problems for the infrastructure required for storage and analysis [9]. The size of data involved strains network capabilities as well as storage and compute power. Inequality in the ability to address either issue will again have impacts on HD. Data filtration could alleviate the size issue but would cause undesirable information loss. To retain as much relevant data as possible, special-purpose computer clusters can be built to withstand the required load. This often leads to centralization of data into a single location [10], improving safety but impeding accessibility and potentially affordability. Inversely, distributed infrastructure, combining compute and storage, is being explored to keep the data close to its source, thus minimizing the transport of data, maximizing its proximity to expert analysts and safe-guarding its access. Furthermore, this can allow for a more flexible and affordable infrastructure that is better suited to a broader range of environments, reflecting the diversity underlying HD. Solutions must be constantly explored to enable effective, high-throughput analyses without compromising data integrity, safety, and accessibility.

**Figure:**
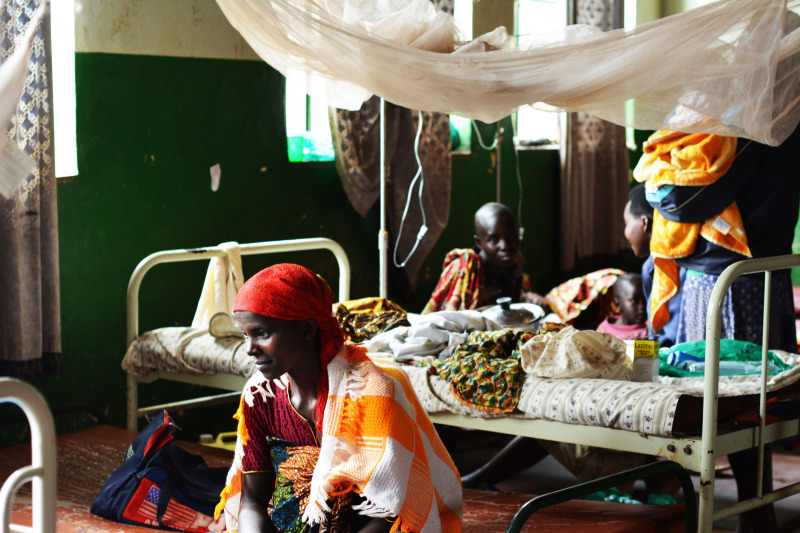
Photo: The authors acknowledge www.pixabay.com artist elisemertens89.

## FUTURE OUTLOOK

In conclusion, technological progress can provide scientists with abundant information at various scales, and improved infrastructure to tackle the issue of HD. However, information is not necessarily aggregated and freely available, also the increased amount and diversity of data does not automatically translate into actionable improvements in population health, risk reduction, diagnosis, and treatment. If society wants to properly address HD, then all stakeholders - the diverse communities, clinicians, scientists, engineers, and policymakers - must work together at every stage of the process; from the identification of a need, the experimental design, the data generation, and analysis to the dissemination and implementation of outcomes.
